# Effect of climatic factors on the seasonal fluctuation of human brucellosis in Yulin, northern China

**DOI:** 10.1186/s12889-020-08599-4

**Published:** 2020-04-16

**Authors:** Kun Liu, Zurong Yang, Weifeng Liang, Tianci Guo, Yong Long, Zhongjun Shao

**Affiliations:** 1 Department of Epidemiology, Ministry of Education Key Lab of Hazard Assessment and Control in Special Operational Environment, School of Public Health, Air Force Medical University, Xi’an, 710032 China; 2 Health Commission of Shaanxi Province, Xi’an, 710003 China

**Keywords:** Human brucellosis, Climatic factors, Granger causality test, Distributed lag non-linear models, Quantitative associations

## Abstract

**Background:**

Brucellosis is a serious public health problem primarily affecting livestock workers. The strong seasonality of the disease indicates that climatic factors may play important roles in the transmission of the disease. However, the associations between climatic variability and human brucellosis are still poorly understood.

**Methods:**

Data for a 14-year series of human brucellosis cases and seven climatic factors were collected in Yulin City from 2005 to 2018, one of the most endemic areas in northern China. Using cross-correlation analysis, the Granger causality test, and a distributed lag non-linear model (DLNM), we assessed the quantitative relationships and exposure-lag-response effects between monthly climatic factors and human brucellosis.

**Results:**

A total of 7103 cases of human brucellosis were reported from 2005 to 2018 in Yulin City with a distinct peak between April and July each year. Seasonal fluctuations in the transmission of human brucellosis were significantly affected by temperature, sunshine duration, and evaporation. The effects of climatic factors were non-linear over the 6-month period, and higher values of these factors usually increased disease incidence. The maximum separate relative risk (RR) was 1.36 (95% confidence interval [CI], 1.03–1.81) at a temperature of 17.4 °C, 1.12 (95% CI, 1.03–1.22) with 311 h of sunshine, and 1.18 (95% CI, 0.94–1.48) with 314 mm of evaporation. In addition, the effects of these three climatic factors were cumulative, with the highest RRs of 2.27 (95% CI, 1.09–4.57), 1.54 (95% CI, 1.10–2.18), and 1.27 (95% CI, 0.73–2.14), respectively.

**Conclusions:**

In Yulin, northern China, variations in climatic factors, especially temperature, sunshine duration, and evaporation, contributed significantly to seasonal fluctuations of human brucellosis within 6 months. The key determinants of brucellosis transmission and the identified complex associations are useful references for developing strategies to reduce the disease burden.

## Background

Brucellosis is a highly infectious zoonosis caused by bacteria of the genus *Brucella* spp. [1]. More than half a million new cases of brucellosis are reported in more than 170 countries each year, causing a serious human health burden and significant economic losses in agriculture, especially in developing countries in Asia, sub-Saharan Africa, and Latin America [2]. The disease is characterized by allergic reactions, symptoms with an incubation period of 5 days to 2 months, fever, migratory arthritis, night sweats, body aches, testicular pain and swelling, and weakness [3]. Human infections occur mainly through contact with infected livestock or aborted materials or consumption of unpasteurized food contaminated with *Brucella* spp., especially raw milk and other products from goat and sheep [4, 5]. People of all ages and both sexes can be affected by the disease, and the occupational groups at higher risk of infection are shepherds, slaughterhouse workers, animal breeders, and veterinarians [3]. A field study reported that 79.4% of human infections occurred in individuals regularly in contact with domestic animals [6]. Given that *Brucella* spp. infections occur via various routes of diverse animal reservoirs and transmissions as well as the lack of effective vaccines and timely treatment, the rates of initial treatment failure and relapse along with the degree of substantial residual disability are high [6, 7].

In China, human brucellosis was first reported in Shanghai in 1905, and reporting to health authorities became mandatory in 1955 [8]. From 1955 to 2014, a total of 513,034 cases of human brucellosis and 170 deaths were registered, and 346,682 (67.6%) cases were reported in the recent period from 2004 to 2014 [3]. In view of the fast-growing demand for milk and meat products, the incidence of brucellosis and the number of endemic areas have increased dramatically in China since 1999. To date, brucellosis is endemic in 30 of 32 provinces or autonomous regions of China, and the high-risk regions are mainly located in northern China, including the Inner Mongolia Autonomous Region and neighboring provinces [7, 9].

Outbreaks of human brucellosis in China usually have an obvious seasonal fluctuation and occur primarily between February and July [5, 9]. Previous studies showed that the seasonality of brucellosis might be linked to human activities and ecological factors, especially climatic variability [10, 11]. Li et al. found that temporal peaks in the incidence of brucellosis were strongly associated with lower temperatures and less sunshine in the winter and spring [7]. Therefore, we hypothesized that climatic factors likely influence the ecology of brucellosis both directly and indirectly by affecting several parameters, including the growth and reproduction dynamics of domestic animals, interactions between sheep/goats and humans, pathogen replication, and population immunity [7, 10–12]. However, few studies have investigated the effects of climatic factors on this important zoonosis. In light of the growing burden of brucellosis, understanding the quantitative relationships between climatic factors and the seasonality of brucellosis and providing early warning of disease epidemics through climate forecasting are critical needs.

In the current study, we selected one of the regions most severely affected by brucellosis in northern China. Data for a 14-year series were collected monthly, and mathematical models were adopted to evaluate exposure-lag-response effects and quantitative relationships between climatic variability and the incidence of human brucellosis.

## Materials and methods

### Study area

Yulin is a prefecture-level city of Shaanxi Province in northern China, bordered by the brucellosis high-prevalence provinces of Inner Mongolia, Shanxi, Gansu, and Ningxia, with a spatial extent of 36°57′-39°34′N and 107°28′-115°15′E (Fig. S1). It consists of 2 districts and 10 counties with area of 43,578 km^2^ and a total population of 3,382,000 in 2016. The region belongs to a part of the transitional landscape spanning from the Maowusu Desert to the Loess Plateau and is a typical crisscross zone of animal husbandry and farming. The climate of the region is typical of northern China, i.e., temperate arid and semi-arid with continental monsoons, cold and long winters, hot and slightly humid summers (http://www.yl.gov.cn/).

### Data collection

In China, human brucellosis is considered a notifiable category B infectious disease, and human cases must be reported to the local Center for Disease Control and Prevention (CDC). A confirmed case of brucellosis is diagnosed based on a combination of epidemiologic exposure, clinical signs, and verification of infection by serological tests, including the standard plate agglutination test and/or rose bengal plate test and/or serum agglutination test, or isolation of *Brucella* spp. according to the guidelines of the World Health Organization. In this study, consecutive data of confirmed brucellosis cases from January 1, 2005 to December 31, 2018 were collected from the CDC of Shaanxi Province, and demographic data were obtained from the Sixth National Census in 2010. Data on seven local climatic variables—temperature, precipitation, relative humidity, sunshine duration, evaporation, atmospheric pressure, and wind velocity—were collected daily during the study period by the Chinese Bureau of Meteorology (http://data.cma.cn/).

### Statistical analysis

Considering the incubation period of human brucellosis and the data stability, a monthly time-series analysis was conducted to determine the temporal associations between climatic factors and the incidence of human brucellosis. First, climatic factors and the incidence rates of human brucellosis were described, and a cross-correlation analysis was performed to assess correlations. Next, a Granger causality test was performed for each climatic factor to determine the likely effect of climate variability on the transmission of human brucellosis. Final, the variables selected in the Granger causality test were included in a distributed lag non-linear model (DLNM) to examine their non-linear and lagged effects on the disease transmission. The methodology is based on the definition of a cross-basis, which is a function expressed by the combination of two sets of basic functions that specify the relationships in the dimension of predictor and time lags, respectively [13, 14]. The structure of the DLNM was as follows:

$$ {Y}_t\sim P\mathrm{oisson}\ \left({\upmu}_t\right) $$

$$ \log \left(\mathrm{E}\left({\mathrm{Y}}_t\right)\right)=\alpha +\beta {T}_{t,l}+ NS(time)+ month $$

where t is the month of the observation; *Y*_*t*_ is the observed incidence of brucellosis in month t; *α* is the intercept; *T*_*t*, *l*_ is a matrix obtained by applying the DLNM to climatic factors, β is the vector of coefficients for *T*_*t*, *l*_, and *l* is the time lag. Four degrees of freedom per year were used to adequately control for the seasonality of brucellosis transmission. *Month* is an ordinal variable for the month of the year. A spline–natural cubic spline for climatic factors that generated a basis matrix of polynomials was used to simulate the non-linear effect and the lag effect. The average value for each variable was defined as the baseline reference for calculating the RR, and the separate effect (in a specific lag month) and cumulative effect (in all months preceding a specific lag month) on the incidence of brucellosis were calculated.

The analyses were performed using R software version 3.5.1 with the packages “lmtest” and “dlnm”. All statistical tests were two-sided, and a *p* value < 0.05 was considered statistically significant.

### Ethical statement

The National Health Commission of China determined whether ethical approval was required for this study. In China, the collection of data from human brucellosis cases was part of routine public health surveillance, and such data collection was exempt from institutional review board assessment [15, 16].

## Results

### Temporal trend and seasonality of human brucellosis and climatic factors

**Fig. 1:**
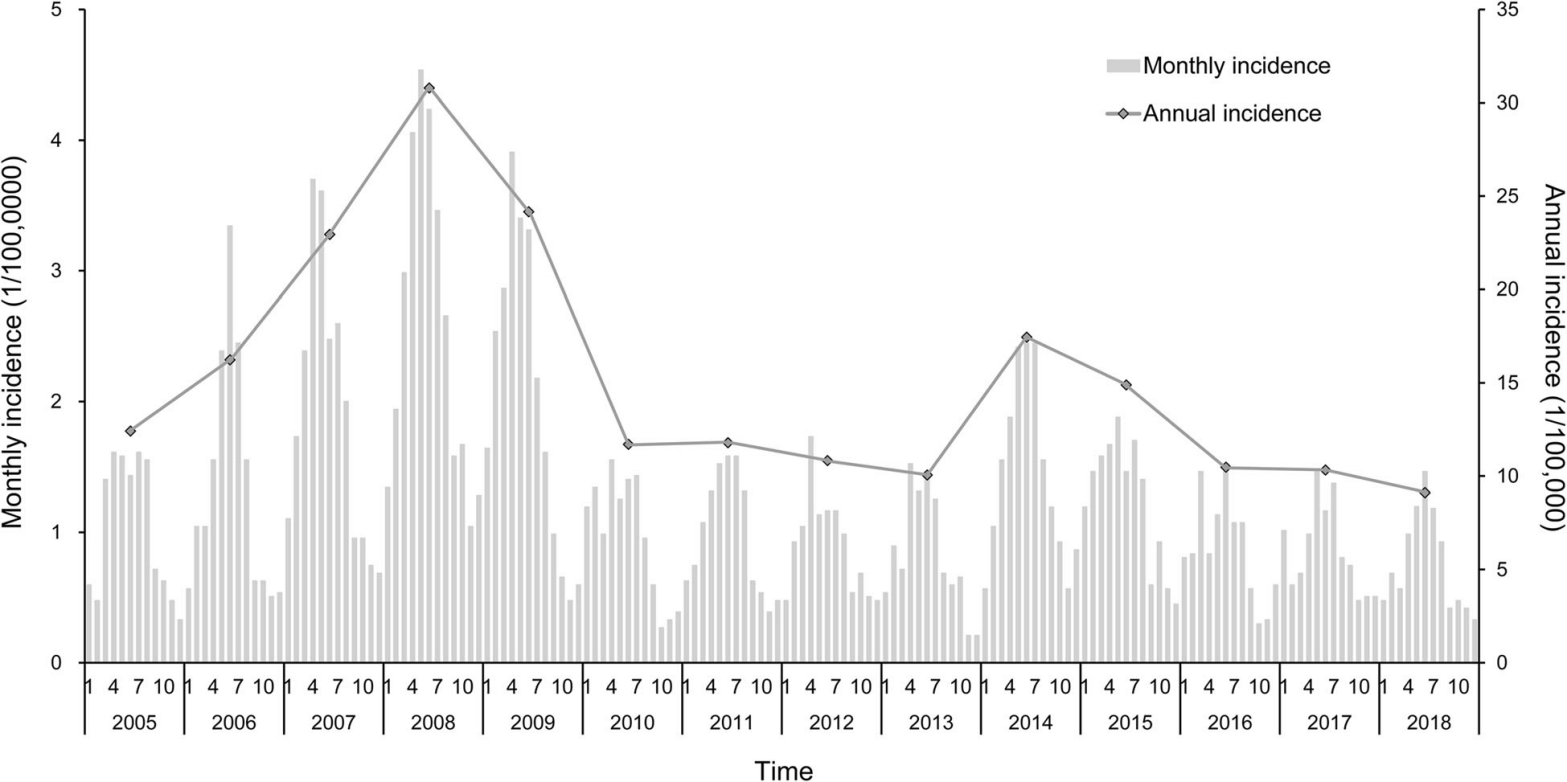
Epidemic curve of the incidence of human brucellosis in Yulin City, Northern China, 2005–2018

**Fig. 2:**
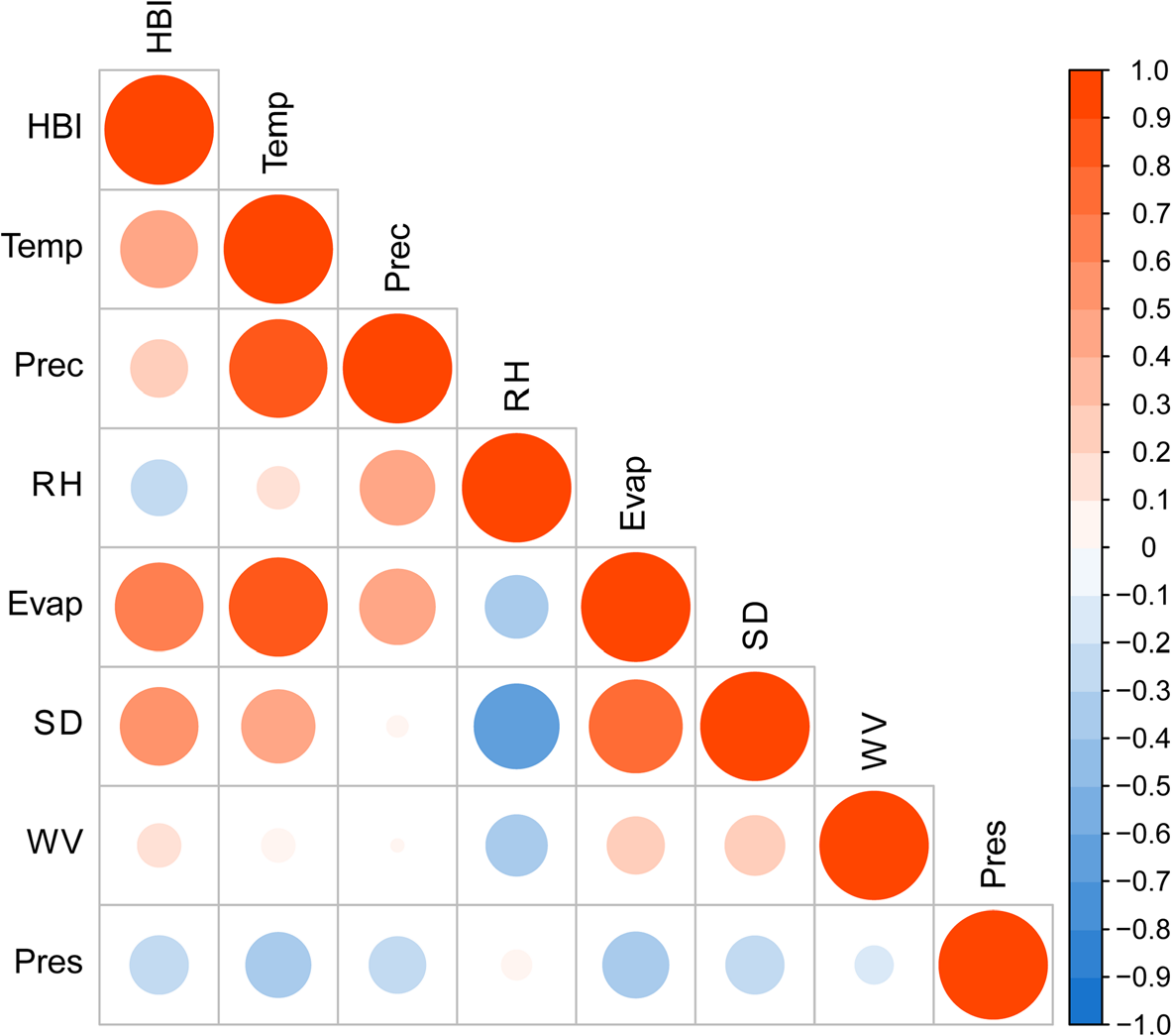
Cross correlation coefficients between climatic factors and the incidence of human brucellosis in Yulin City, Northern China, 2005–2018. (IHB: monthly incidence of human brucellosis; Temp: monthly mean temperature; Prec: monthly mean precipitation; Evap: monthly cumulative evaporation; SD: monthly cumulative sunshine duration; RH: monthly mean relative humidity; Pres: monthly mean atmospheric pressure; WV: monthly mean wind velocity)

In the period from 2005 to 2018, a total of 7103 cases of human brucellosis were reported in the study area. The annual average incidence of the disease was 15.24 per 100,000 persons, and the highest rate was 30.79/100,000 in 2008. The epidemic curves revealed a seasonal peak in incidence from April to July, accounting for 51.33% of all cases. In addition, the mean and standard deviation of the monthly incidence was 1.27 ± 0.86 per 100,000, and the incidence was highest in May (Fig. 1). Summary statistics for the climatic factors in the study period are shown in Table 1. The cross-correlation analysis revealed that five climatic factors—temperature, precipitation, evaporation, sunshine duration, and wind velocity—were positively correlated with the human brucellosis incidence, and relative humidity and atmospheric pressure were negatively associated with the disease incidence (Fig. 2 and Table S1). The results of the Granger causality tests indicated that the temporal distribution of human brucellosis was strongly affected by temperature, sunshine duration, and evaporation (Table 2).

**Table 1:** Descriptive statistics of monthly climatic variables in Yulin City, Northern China, 2005–2018

Variables	Min	P_25_	P_50_	P_75_	Max	Mean ± SD
Temperature (°C)	−11.88	0.29	11.17	19.41	25.45	9.68 ± 10.83
Precipitation (mm)	0	3.99	19.34	53.54	198.80	36.67 ± 42.19
Relative humidity (%)	23.99	42.38	50.46	62.57	77.80	51.69 ± 12.60
Sunshine duration (h)	119.30	201.74	227.78	259.98	311.30	228.34 ± 72.23
Evaporation (mm)	25.55	68.80	128.32	188.93	314.07	135.63 ± 40.96
Atmospheric pressure (Pa)	876.55	882.73	887.52	890.74	896.79	886.72 ± 13.64
Wind velocity (m/s)	1.59	2.06	2.38	2.74	3.40	2.40 ± 0.43

Min: minimum level of the variable; P_25_: 25th percentile of the variable; P_50_: 50th percentile of the variable; P_75_: 75th percentile of the variable; Max: maximum level of the variable; SD: standard deviation

**Table 2:** Granger causality tests for climatic variables and the monthly incidence of human brucellosis in Yulin City, Northern China, 2005–2018

	Temperature	Precipitation	Relative humidity	Sunshine duration	Evaporation	Atmospheric pressure	Wind velocity
F-statistics	2.358	1.439	0.827	3.625	2.579	3.741	3.520
*P*-value	0.033*	0.204	0.551	0.002*	0.021*	0.067	0.769

*P < 0.05

### Non-linear and lagged effects of climatic factors on the incidence of human brucellosis

#### Temperature

**Fig. 3:**
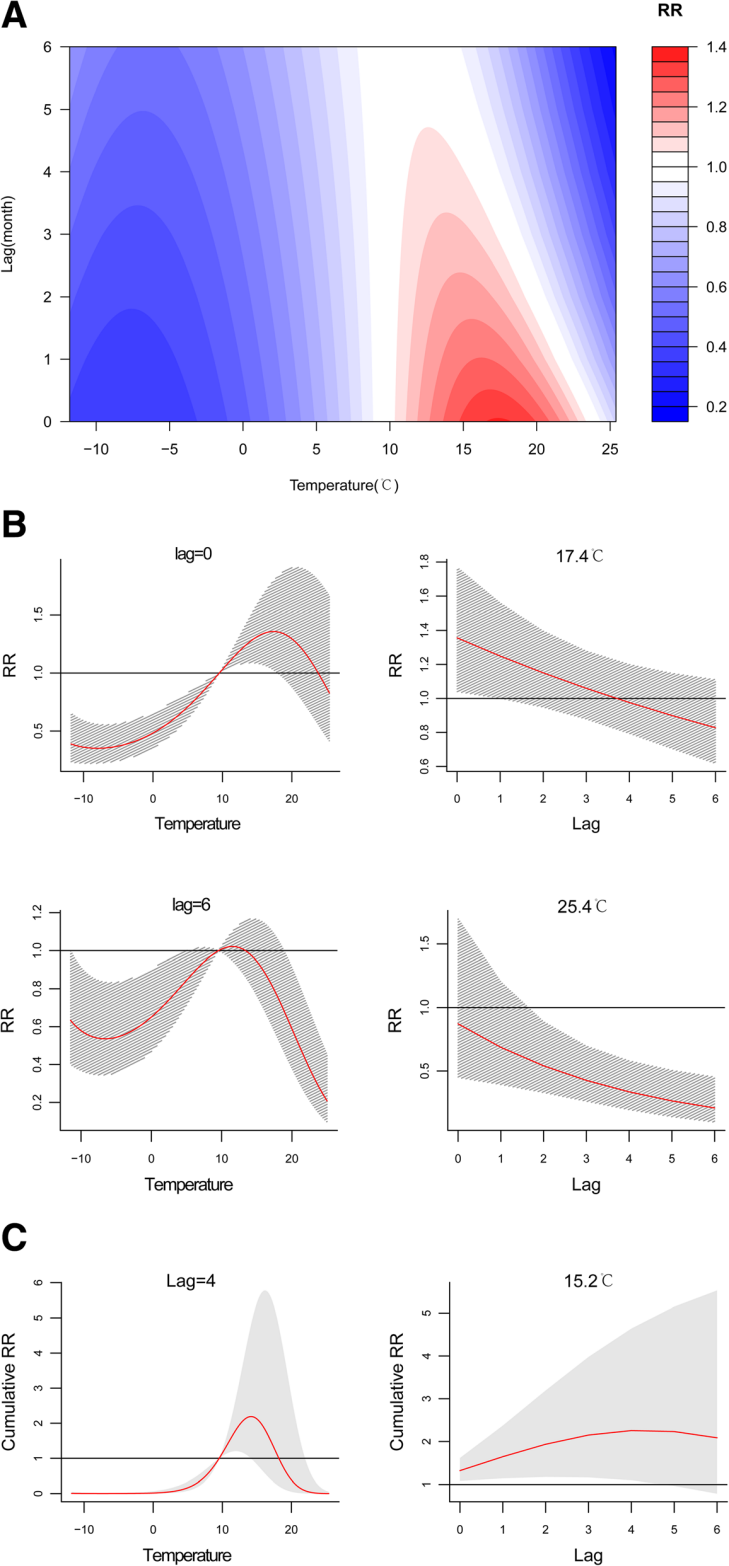
**a** Contour plots of the combined effect of time lags and temperature on the relative risk of transmission of human brucellosis. **b**. Effect of specific temperatures and time lags on the relative risk of transmission of human brucellosis. The red lines indicate the mean relative risk, and the gray lines are 95% CI. **c**. Effect of specific temperatures and time lags on the cumulative risk of transmission of human brucellosis. The red lines indicate the mean relative risk, and the gray areas correspond to 95% CI

A comprehensive summary of the non-linear relationship between monthly average temperature and the incidence of human brucellosis over a 6-month period is shown in Fig. 3a. The RR of human brucellosis increased significantly for 3 to 4 months as the temperature increased (Figure S2). The separate effects of different temperatures and two time lags (0 and 6 months) on the RRs together with 95% confidence intervals (95% CI) are shown in Fig. 3b. The association presented an inverted U-shaped function, i.e., as temperature increased, the RR values increased to a peak value and then decreased. The maximum RR was 1.36 (95% CI, 1.03–1.81) at approximately 17.4 °C in the current month (time lag 0), and the minimum RR was 0.19 (95% CI, 0.08–0.42) at approximately 25.4 °C at time lag 6 (Table 3). The effect of temperature on the cumulative risk of disease is shown in Fig. 3c. Higher temperatures increased the cumulative risk from time lag 0 to 4, and the RR of 2.27 (95% CI, 1.09–4.57) was highest at approximately 15.2 °C in month 4 sss.

**Table 3:** DNLM model results for separate and cumulative effects of monthly climatic variables on the relative risk of human brucellosis in Yulin City, Northern China, 2005–2018

Variable	Separate effect						Cumulative effect		
	Maximum RR (95% CI)	Variable value	Lag month	Minimum RR (95% CI)	Variable value	Lag month	Maximum RR (95% CI)	Variable value	Lag month
Temperature (°C)	1.36 (1.03–1.81)	17.4 °C	0	0.19 (0.08–0.42)	25.4 °C	6	2.27 (1.09–4.57)	15.2 °C	4
Sunshine duration (h)	1.12 (1.03–1.22)	311 h	0	0.86 (0.78–0.96)	121 h	0	1.54 (1.10–2.18)	311 h	6
Evaporation (mm)	1.18 (0.94–1.48)	314 mm	0	0.67 (0.50–10.91)	314 mm	6	1.27 (0.73–2.14)	314 mm	2

#### Duration of sunshine

**Fig. 4:**
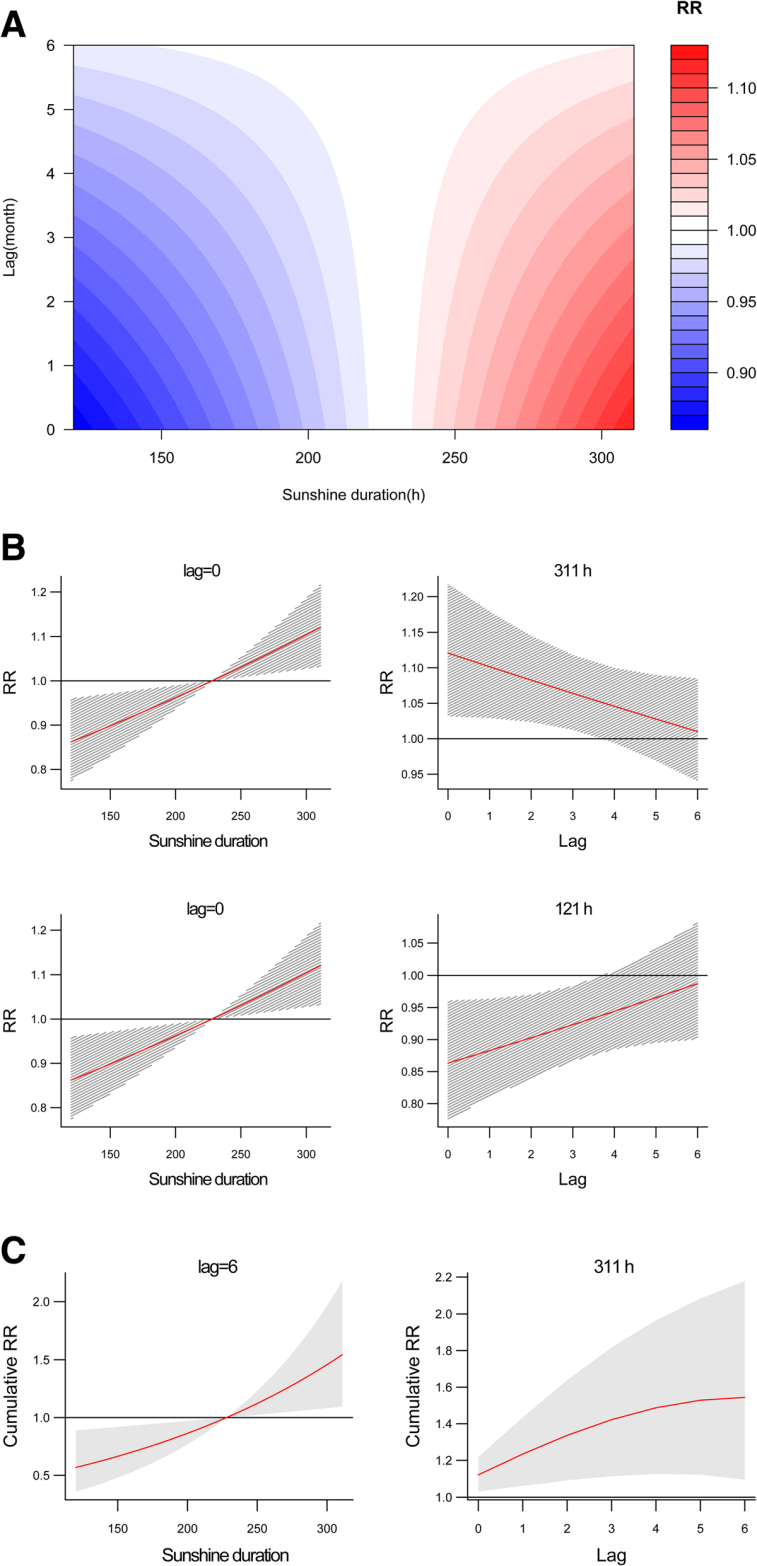
**a** Contour plots of the combined effect of time lags and sunshine duration on the relative risk of transmission of human brucellosis. **b**. Effect of specific sunshine durations and time lags on the relative risk of transmission of human brucellosis. The red lines are the mean relative risks, and the gray lines are 95% CI. **c**. Effects of specific sunshine durations and time lags on the cumulative risk of transmission of human brucellosis. The red lines are the mean relative risks, and the gray areas are 95% CI

The combined effect of time lag and duration of sunshine on the RR of human brucellosis is shown in Fig. 4a. A longer sunshine duration significantly increased the RR for all lag times compared with the average duration, whereas a shorter sunshine duration decreased the RR (Figure S3). The effects of sunshine duration were strongest in time lag 0, with a maximum RR of 1.12 (95% CI, 1.03–1.22) at 311 h and a minimum RR of 0.86 (95% CI, 0.78–0.96) at 121 h (Fig. 4b and Table 3). There was a significantly positive association between sunshine duration and cumulative RR for all time lags, and the effect was higher as the sunshine duration increased. The cumulative RR was highest of 1.54 (95% CI, 1.10–2.18) at 311 h with time lag 6 (Fig. 4c and Table 3).

#### Evaporation

**Fig. 5:**
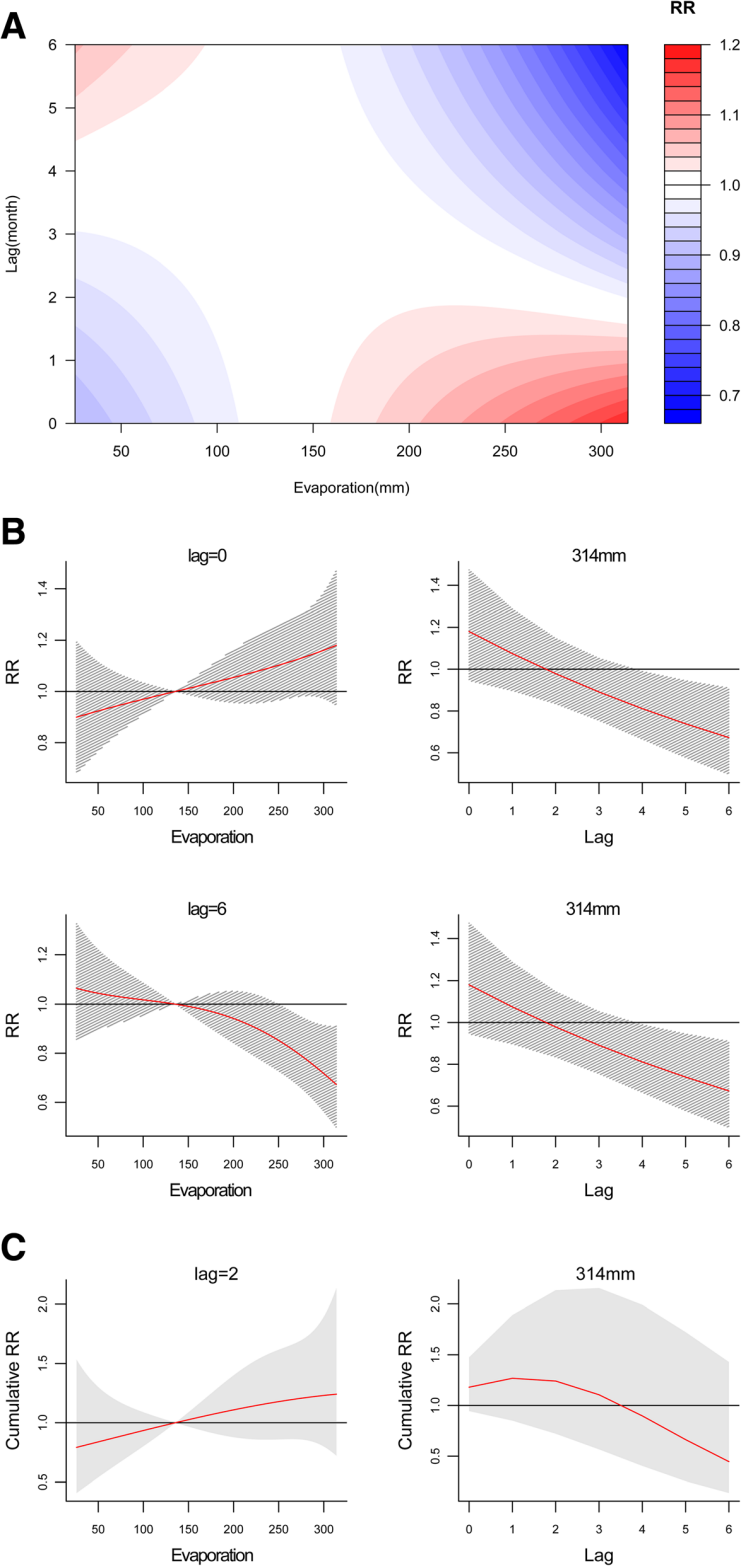
**a**. Contour plots of the combined effect of evaporation and time lags on the relative risk of transmission of human brucellosis. **c**. Effects of specific evaporations and time lags on the relative risk of transmission of human brucellosis. The red lines are the mean relative risks, and the gray lines are 95% CI. **c**. Effect of specific evaporations and time lags on the cumulative risk of transmission of human brucellosis. The red lines are the mean relative risks, and the gray areas are 95% CI

The combined effect of time lags and evaporation on the RR of human brucellosis is shown in Fig. 5a. Higher evaporation increased the RR in the short terms (within 2 months) and then decreased over time. In contrast, lower evaporation decreased the RR in the short terms (within 2 months) and increased the RR over time (Figure S4). The variations in RR at 314 mm were biggest, with a maximum RR of 1.18 (95% CI, 0.94–1.48) in time lag 0 and a minimum RR of 0.67 (95% CI, 0.50–0.10.91) in time lag 6 (Fig. 5b and Table 3). The cumulative RR of 1.27 (95% CI, 0.73–2.14) was highest at approximately 314 mm (Fig. 5c and Table 3).

## Discussion

Human brucellosis is a serious public health problem in the global livestock husbandry areas, and the seasonal distribution of the disease is strongly associated with climate variability [2, 7]. The critical findings of our study were that temperature, sunshine duration, and evaporation were key determinants of disease seasonality in Yulin, and similar associations could easily be expanded over wider regions in northern China. Meanwhile, the lagged effects of climatic factors could provide sufficient time to develop targeted interventions for decreasing the disease burden in humans and livestock.

Seasonal climatic conditions may affect multiple aspects of climate-sensitive infectious diseases [17]. Consequently, climatic factors can be used to forecast the occurrence of disease outbreaks within periods of weeks to months [18]. Public health professionals use mathematical models to investigate the complex relationships between disease epidemics and climate data [19]. The DLNM was conducted in the study to examine the quantitative relationships and exposure-lag-response effects between climate variability and human brucellosis. This model has been effectively adopted to assess the non-linear relationships between meteorological factors and infectious diseases such as dengue, malaria, hand-foot-mouth disease, tuberculosis, and mumps [20–25].

The results of the present study suggest that changes in temperature may have a greater impact on the epidemics of human brucellosis than other climatic factors. The increasing temperature increased *Brucella* spp. development and replication in the host and increased the frequency of exposure of susceptible animals and humans [7]. Therefore, the transmission and persistence of *Brucella* spp. may be enhanced in warmer conditions, as demonstrated in our study, in which the maximum cumulative effect was highest at 15.2 °C in 4 months. In this respect, higher temperatures in late spring and early summer increase husbandry activities for sheep and goats, including shearing, breeding, processing of meat products, and commercialization of sheep products, consequently increasing the exposure of susceptible animals to contaminated animal products [11, 26]. In contrast, the RR of brucellosis transmission decreases during winter because colder temperatures may limit the development of infective organisms [27]. In addition, Yulin is a region known for cashmere production and sheep farming. Meanwhile, the quarantine measures in sheep farms, adequate management of animal abortion products, and human protective measures are all deficient, causing more people to be exposed to the contaminated animal products [28, 29].

The variability in monthly cumulative evaporation and sunshine strongly affects the transmission of *Brucella* spp., which develops intracellularly in the host and outside the host in dust, soil, and water [11, 26, 27]. The high incidence of brucellosis may be partly due to sheep breeding and abortion in subsequent lambing [7, 23]. Considering that the gestation period in sheep is 6 months, our results suggest that the increased sunshine duration in early winter could promote estrus in sheep, resulting in the birth of animals more susceptible to infections with the adoption of high-risk breeding activities [12]. In addition, higher levels of evaporation and sunshine may favor drought, limiting plant germination. Meanwhile, dry environments may cause human skin dryness and fissures, increasing the exposure risk. As for China, the national policy of converting farmland into forests has limited field grazing, and breeding methods for sheep and goats have been adapted to raising livestock in pens [28]. However, the levels of minerals in stored grasses cultivated are unbalanced, making sheep and goats immunologically compromised and more susceptible to diseases [29].

This study aimed to investigate the effects of climatic factors on the incidence of human brucellosis, and several limitations should be acknowledged. First, the data were obtained from a passive surveillance system, in which some cases of human brucellosis might not have been reported because of milder clinical symptoms or delayed reporting in rural areas [30]. Second, the temporal dynamics of brucellosis are affected by non-climatic factors as well, including the number of affected sheep, immunity of the local population, human activities and movements, commercialization of sheep products, and eating habits. The analytical capability of model may be limited because of omitted covariates or changes in environmental and human factors. These limitations should be further investigated.

## Conclusion

The present study provides evidence for a strong relationship between climatic factors and the incidence of human brucellosis in Yulin City, and the results can be replicated easily in livestock husbandry areas with similar environmental conditions. The key determinants of brucellosis transmission and the identified complex associations are useful references for the development of strategies for early warning, prevention, and control of seasonal epidemics.

## Supplementary information

**Additional file 1: Table S1.** Cross correlation coefficients between monthly human brucellosis incidence and climatic variables in Yulin City, the Northern China, 2005-2018. **Figure S1.** Study areas in China. The map was created by Kun Liu in ArcGIS 10.2 Software, ESRI Inc., Redlands, CA, USA, (https://www.arcgis.com/index.html).**Figure S2.** Three-dimensional graph of the relationship between monthly mean temperature and human brucellosis incidence. **Figure S3.** Three-dimensional graph of the relationship between monthly cumulative sunshine duration and human brucellosis incidence. **Figure S4.** Three-dimensional graph of the relationship between monthly cumulative evaporation and human brucellosis incidence.

## Data Availability

The data of brucellosis patients in Yulin city are available from Shaanxi Province Center for Disease Control and Prevention. All data used for analysis are available upon a proper request from the corresponding author Zhongjun Shao at 13759981783@163.com.

## Abbreviations

CDC: Center for Disease Control and Prevention
CI: Confidence interval
DLNM: Distributed lag non-linear model
Evap: Monthly cumulative evaporation
IHB: Monthly incidence of human brucellosis
Prec: Monthly mean precipitation
Pres: Monthly mean atmospheric pressure
RH: Monthly mean relative humidity
RR: Relative risk
SD: Monthly cumulative sunshine duration
Temp: Monthly mean temperature
WV: Monthly mean wind velocity
