# Supplementary material for: Effect of climatic factors on the seasonal fluctuation of human brucellosis in Yulin, northern China
Source: BMC Public Health. 2020 Apr 16;20:506. doi: 10.1186/s12889-020-08599-4 (PMC7164191; doi:10.1186/s12889-020-08599-4)
Supplement: Supplementary file 1 — Additional file 1: Table S1. Cross correlation coefficients between monthly human brucellosis incidence and climatic variables in Yulin City, the Northern China, 2005-2018. Figure S1. Study areas in China. The map was created by Kun Liu in ArcGIS 10.2 Software, ESRI Inc., Redlands, CA, USA, (https://www.arcgis.com/index.html).Figure S2. Three-dimensional graph of the relationship between monthly mean temperature and human brucellosis incidence. Figure S3. Three-dimensional graph of the relationship between monthly cumulative sunshine duration and human brucellosis incidence. Figure S4. Three-dimensional graph of the relationship between monthly cumulative evaporation and human brucellosis incidence. [file 12889_2020_8599_MOESM1_ESM.zip › Additional files-R5 versionR5.docx]

**Additional files:**

**Effect of climatic factors on the seasonal fluctuation of human brucellosis in Yulin, Northern China**

1. **Table S1**
2. **Figure S1-S4**

**Table S1. Cross correlation coefficients between monthly human brucellosis incidence and climatic variables in Yulin City, the Northern China, 2005-2018.**

| Variables | HBI | Temp | Prec | RH | Evap | SD | WV |
| --- | --- | --- | --- | --- | --- | --- | --- |
| HBI | 1 |  |  |  |  |  |  |
| Temp | 0.50* | 1 |  |  |  |  |  |
| Prec | 0.27* | 0.80* | 1 |  |  |  |  |
| RH | -0.26* | 0.15* | 0.47* | 1 |  |  |  |
| Evap | 0.65* | 0.81* | 0.48* | -0.33* | 1 |  |  |
| SD | 0.51* | 0.45* | 0.04 | -0.61* | 0.73* | 1 |  |
| WV | 0.16* | 0.09 | 0.01 | -0.31* | 0.27* | 0.30* | 1 |

HBI: Human brucellosis incidence, Temp: Monthly mean temperature, Prec: Monthly cumulative precipitation, RH: Monthly mean relative humidity, Evap: Monthly cumulative evaporation, SD: Monthly cumulative sunshine duration, WV: Monthly mean wind velocity, Pres: Monthly mean atmospheric pressure.

*: P＜0.05

**Figure Legends**

**Figure S1. Study areas in China.** The map was created by Kun Liu in ArcGIS 10.2 Software, ESRI Inc., Redlands, CA, USA, (https://www.arcgis.com/index.html).

**Figure S2. Three-dimensional graph of the relationship between monthly mean temperature and human brucellosis incidence.**

**Figure S3. Three-dimensional graph of the relationship between monthly cumulative sunshine duration and human brucellosis incidence.**

**Figure S4. Three-dimensional graph of the relationship between monthly cumulative evaporation and human brucellosis incidence.**
