# Supplementary figures and images for: Effect of climatic factors on the seasonal fluctuation of human brucellosis in Yulin, northern China
Source: BMC Public Health. 2020 Apr 16;20:506. doi: 10.1186/s12889-020-08599-4 (PMC7164191; doi:10.1186/s12889-020-08599-4)

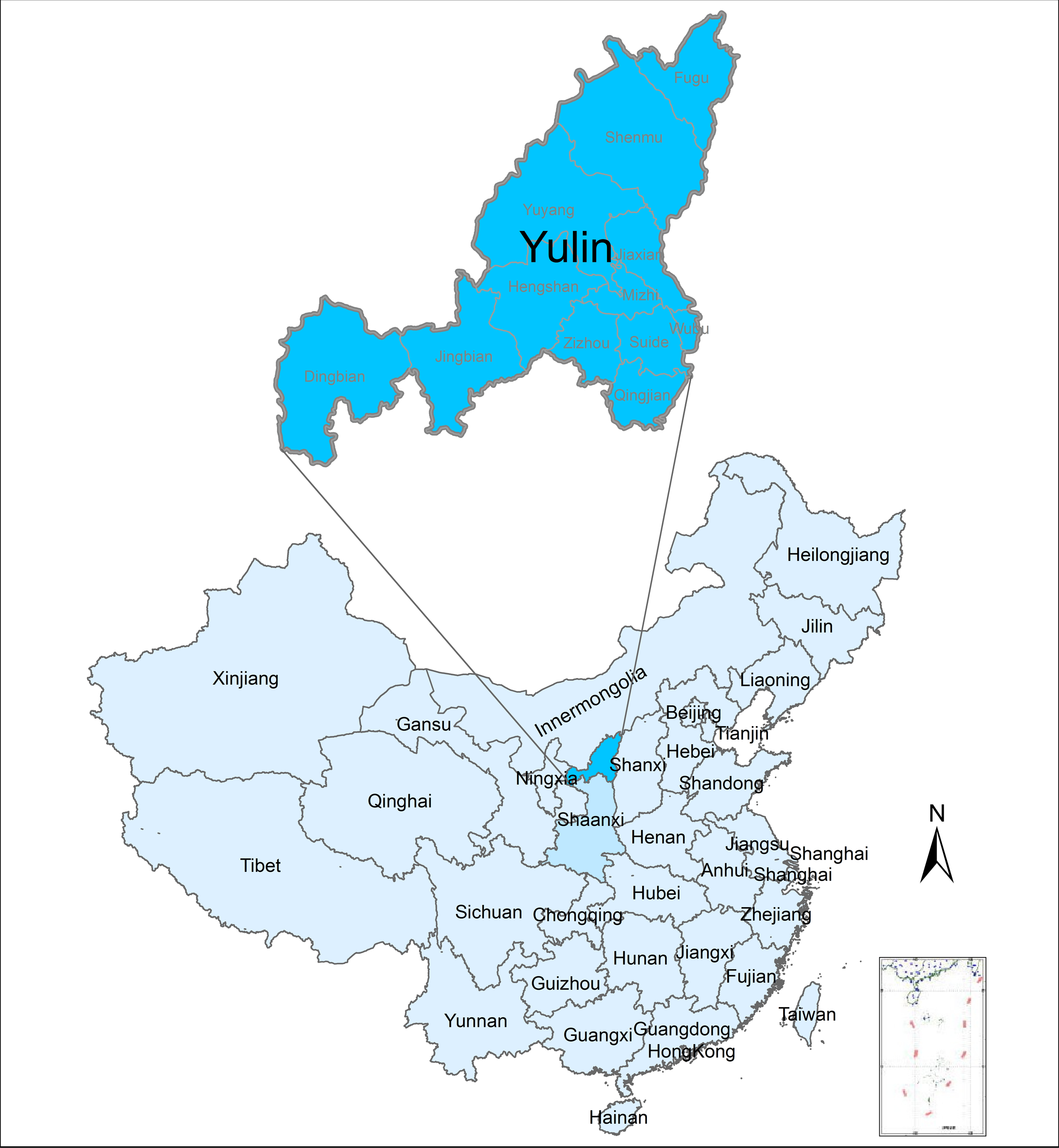

Supplement: Supplementary file 1 — Additional file 1: Table S1. Cross correlation coefficients between monthly human brucellosis incidence and climatic variables in Yulin City, the Northern China, 2005-2018. Figure S1. Study areas in China. The map was created by Kun Liu in ArcGIS 10.2 Software, ESRI Inc., Redlands, CA, USA, (https://www.arcgis.com/index.html).Figure S2. Three-dimensional graph of the relationship between monthly mean temperature and human brucellosis incidence. Figure S3. Three-dimensional graph of the relationship between monthly cumulative sunshine duration and human brucellosis incidence. Figure S4. Three-dimensional graph of the relationship between monthly cumulative evaporation and human brucellosis incidence. [file 12889_2020_8599_MOESM1_ESM.zip › Figure S1R5.tif]

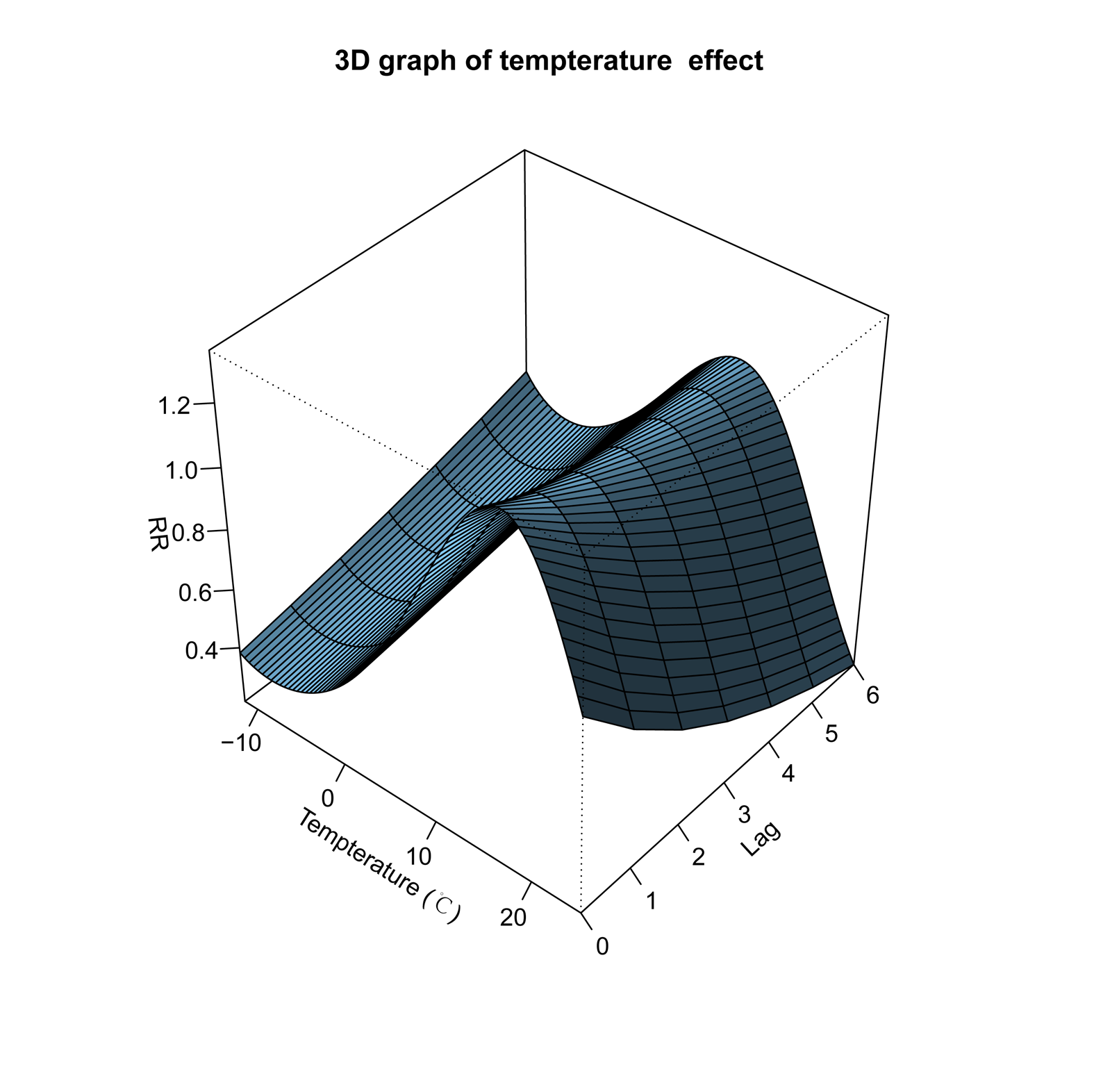

Supplement: Supplementary file 1 — Additional file 1: Table S1. Cross correlation coefficients between monthly human brucellosis incidence and climatic variables in Yulin City, the Northern China, 2005-2018. Figure S1. Study areas in China. The map was created by Kun Liu in ArcGIS 10.2 Software, ESRI Inc., Redlands, CA, USA, (https://www.arcgis.com/index.html).Figure S2. Three-dimensional graph of the relationship between monthly mean temperature and human brucellosis incidence. Figure S3. Three-dimensional graph of the relationship between monthly cumulative sunshine duration and human brucellosis incidence. Figure S4. Three-dimensional graph of the relationship between monthly cumulative evaporation and human brucellosis incidence. [file 12889_2020_8599_MOESM1_ESM.zip › Figure S2R5.tif]

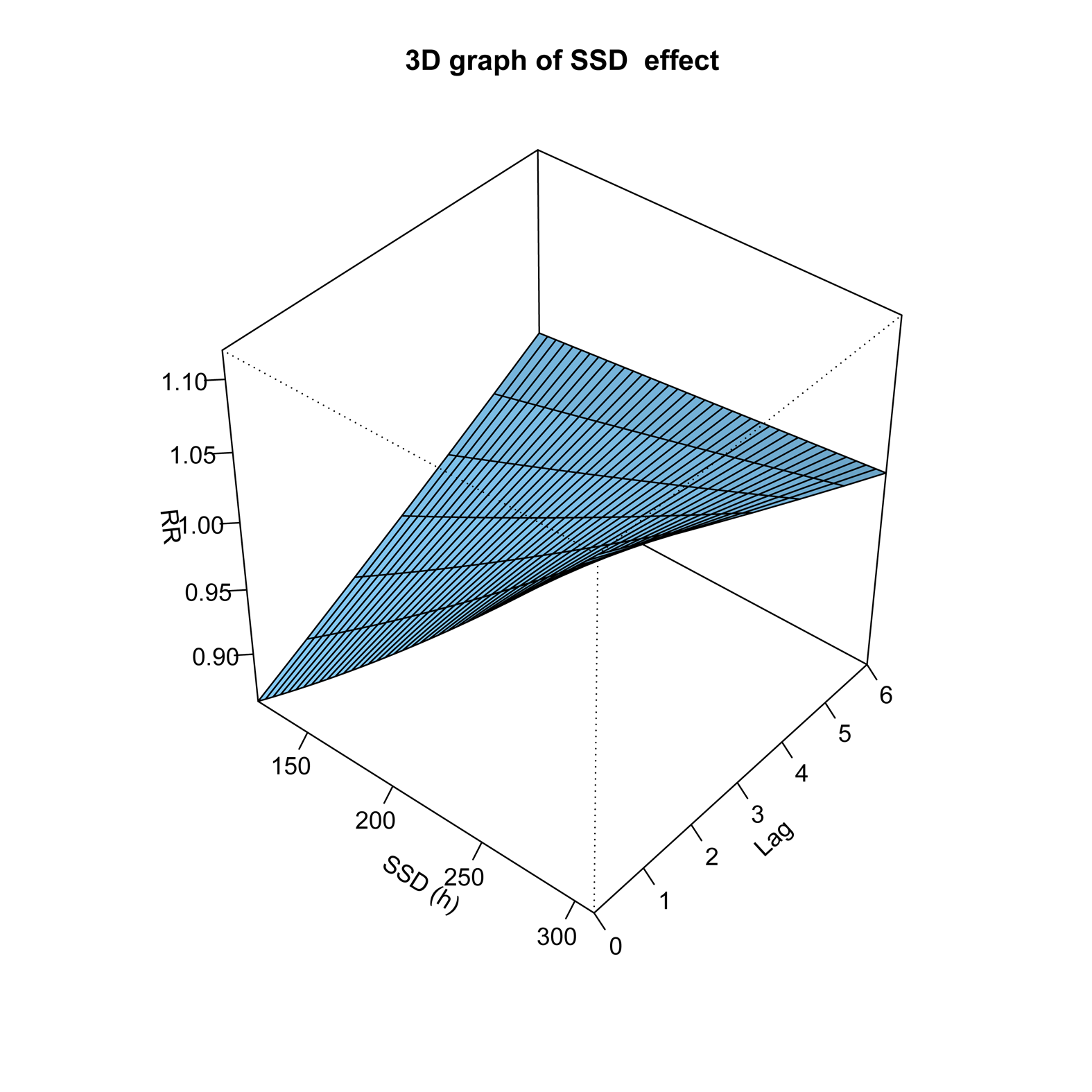

Supplement: Supplementary file 1 — Additional file 1: Table S1. Cross correlation coefficients between monthly human brucellosis incidence and climatic variables in Yulin City, the Northern China, 2005-2018. Figure S1. Study areas in China. The map was created by Kun Liu in ArcGIS 10.2 Software, ESRI Inc., Redlands, CA, USA, (https://www.arcgis.com/index.html).Figure S2. Three-dimensional graph of the relationship between monthly mean temperature and human brucellosis incidence. Figure S3. Three-dimensional graph of the relationship between monthly cumulative sunshine duration and human brucellosis incidence. Figure S4. Three-dimensional graph of the relationship between monthly cumulative evaporation and human brucellosis incidence. [file 12889_2020_8599_MOESM1_ESM.zip › Figure S3R5.tif]

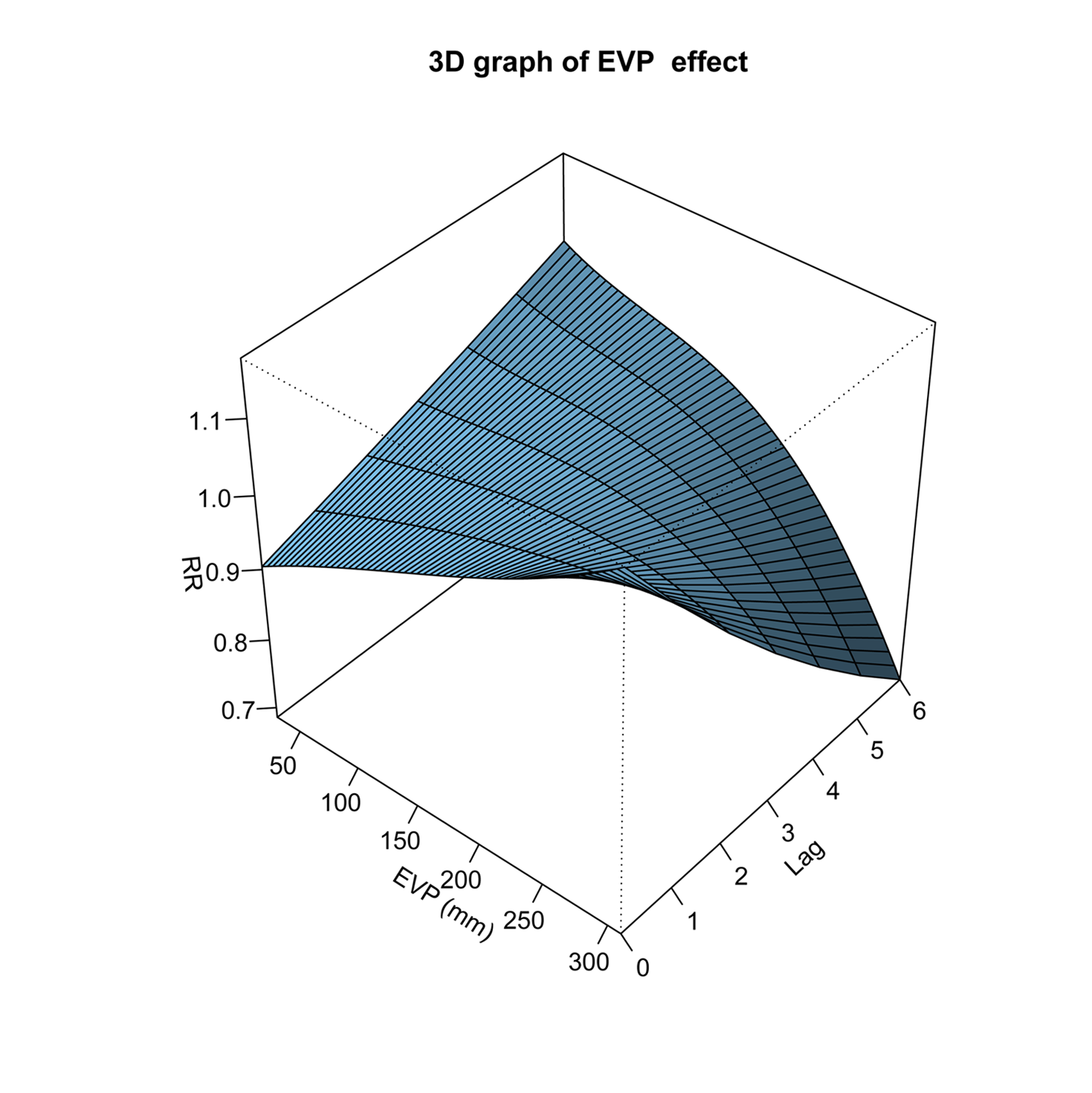

Supplement: Supplementary file 1 — Additional file 1: Table S1. Cross correlation coefficients between monthly human brucellosis incidence and climatic variables in Yulin City, the Northern China, 2005-2018. Figure S1. Study areas in China. The map was created by Kun Liu in ArcGIS 10.2 Software, ESRI Inc., Redlands, CA, USA, (https://www.arcgis.com/index.html).Figure S2. Three-dimensional graph of the relationship between monthly mean temperature and human brucellosis incidence. Figure S3. Three-dimensional graph of the relationship between monthly cumulative sunshine duration and human brucellosis incidence. Figure S4. Three-dimensional graph of the relationship between monthly cumulative evaporation and human brucellosis incidence. [file 12889_2020_8599_MOESM1_ESM.zip › Figure S4R5.tif]
